# Cancer testis antigen PRAME: An anti‐cancer target with immunomodulatory potential

**DOI:** 10.1111/jcmm.16967

**Published:** 2021-10-06

**Authors:** Adviti Naik, Remy Thomas, Ghaneya Al‐Khadairi, Rim Bacha, Wouter Hendrickx, Julie Decock

**Affiliations:** ^1^ Translational Cancer and Immunity Center Qatar Biomedical Research Institute (QBRI) Hamad Bin Khalifa University (HBKU) Qatar Foundation (QF) Doha Qatar; ^2^ College of Health and Life Sciences (CHLS) Hamad Bin Khalifa University (HBKU) Qatar Foundation (QF) Doha Qatar; ^3^ Cancer Department, Research Branch Sidra Medicine Doha Qatar

**Keywords:** breast cancer, immune activation, immune checkpoints, immunotherapy, PRAME

## Abstract

PReferentially expressed Antigen in Melanoma (PRAME) is a cancer testis antigen with restricted expression in somatic tissues and re‐expression in poor prognostic solid tumours. PRAME has been extensively investigated as a target for immunotherapy, however, its role in modulating the anti‐tumour immune response remains largely unknown. Here, we show that PRAME tumour expression is associated with worse survival in the TCGA breast cancer cohort, particularly in immune‐unfavourable tumours. Using direct and indirect co‐culture models, we found that PRAME overexpressing MDA‐MB‐468 breast cancer cells inhibit T cell activation and cytolytic potential, which could be partly restored by silencing of *PRAME*. Furthermore, silencing of *PRAME* reduced expression of several immune checkpoints and their ligands, including PD‐1, LAG3, PD‐L1, CD86, Gal‐9 and VISTA. Interestingly, silencing of *PRAME* induced cancer cell killing to levels similar to anti‐PD‐L1 atezolizumab treatment. Comprehensive analysis of soluble inflammatory mediators and cancer cell expression of immune‐related genes showed that PRAME tumour expression can suppress the expression and secretion of multiple pro‐inflammatory cytokines, and mediators of T cell activation, differentiation and cytolysis. Together, our data indicate that targeting of PRAME offers a potential, novel dual therapeutic approach to specifically target tumour cells and regulate immune activation in the tumour microenvironment.

## 1 INTRODUCTION

PReferentially expressed Antigen in Melanoma (PRAME) is a cancer testis antigen (CTA) also known as CT130. It is characterized by restricted expression in germ cells and a low expression in normal somatic tissues such as the testis, epididymis, endometrium, ovaries and adrenal glands.^1^, ^2^ PRAME expression has been demonstrated in a variety of solid and haematological malignancies.^3^, ^4^, ^5^, ^6^ High PRAME tumour expression has been associated with poor prognosis in several solid tumours, increased risk of metastases and shorter disease‐free and overall survival,^7^ whereas it has been found to predict a more favourable outcome in acute myeloid and lymphoblastic leukaemia.^5^, ^8^, ^9^, ^10^, ^11^, ^12^ Several studies suggest that PRAME can induce cell proliferation, reduce cytotoxic drug sensitivity and inhibit apoptosis in a variety of cancers.^13^, ^14^, ^15^, ^16^ The restricted, highly tumour‐specific expression of PRAME in conjunction with its oncogenic functions support the rationale for therapeutic targeting of PRAME in cancer.

PRAME has been extensively investigated as a target for immunotherapy. PRAME tumour expression has been shown to elicit spontaneous humoral and cellular immune responses, and PRAME‐based vaccines and adoptive T cell therapies have shown a favourable safety profile and efficient induction of potent immune responses in tumours.^17^, ^18^, ^19^ The success rate of immunotherapy although varies across tumour types and is, in part, impacted by the inherent ability of cancer cells in shaping an immunosuppressive tumour microenviroment.^20^ Interestingly, PRAME expression in dedifferentiated liposarcoma and leiomyosarcoma tumours was found to be associated with reduced expression of antigen presentation molecules, a common mechanism of immune escape.^21^, ^22^, ^23^, ^24^ In addition, PRAME expression in dedifferentiated liposarcomas has been associated with reduced programmed death ligand‐1 (PD‐L1) expression, a well‐known immune checkpoint ligand that regulates the activity of cytotoxic T lymphocytes through binding to its T cell inhibitory receptor PD‐1.^25^ Immune checkpoint blockade of the PD‐L1/PD‐1 axis that has shown to improve the clinical outcome in numerous cancers including breast cancer.^20^ However, the aforementioned negative correlation between PRAME expression and PD‐L1 expression suggests that treatment with PD‐L1/PD‐1 inhibitors may be less beneficial in PRAME overexpressing tumours as a result of reduced target PD‐L1 expression.

Further evidence suggests that PRAME could be implicated in the regulation of the immune response. For instance, PRAME has been reported to contain 21.8% (iso)leucine residues, and as such likely behaves as a leucine rich repeat (LRR) family protein, sharing structural similarities with Toll‐like receptors (TLR3, TLR4) which play an important role in antimicrobial immune responses.^4^ Moreover, PRAME expression in leukemic cell lines is rapidly induced by signalling pathways that are activated in response to infection and inflammation.^26^ Together, these findings raise the question of how and to what extent PRAME tumour expression could be involved in shaping the anti‐tumour immune response and immunotherapy efficacy.

In the present study, we aimed to investigate the effect of PRAME overexpression in a breast cancer cell line on T cell activity in vitro and on the expression of immune‐related mediators. We show that PRAME expression negatively correlated with overall survival in the TCGA breast cancer cohort, particularly in immune‐unfavourable tumours as defined by the Immunologic Constant of Rejection (ICR) score.^27^ Moreover, we found that PRAME overexpression in breast cancer cells dampens peripheral blood lymphocyte activation and cancer cell killing, concomitant with decreased levels of soluble pro‐inflammatory immune mediators and increased immune checkpoint expression. Our findings suggest that PRAME is a tumour‐associated antigen that can modulate the anti‐tumour immune response.

## 2 MATERIALS & METHODS

### 2.1 Kaplan‐Meier survival analysis and Immune Constant of Rejection (ICR) scoring

Tumour samples from The Cancer Genome Atlas (TCGA) breast cancer dataset were stratified by PRAME expression (cut‐off = median), and survival analysis was visualized using Kaplan‐Meier curves as generated by a modified version of the ggkm function (https://github.com/michaelway/ggkm). Patients with less than one day of follow‐up were excluded and survival data were censored after a follow‐up duration of 10 years. Hazard ratios (HR) between ICR Low and ICR High groups, including corresponding *p*‐values based on chi‐squared test, and confidence interval were calculated using the R package survival.^28^ Immune‐favourable and ‐unfavourable tumours were stratified using ICR clustering as previously described.^29^ Briefly, we performed consensus clustering based on the 20 ICR gene signature using the ConsensusClusterPlus R package.^30^ The cluster with the highest expression of ICR genes was designated as “ICR High”, while the cluster with the lowest ICR gene expression was designated “ICR Low”, and the intermediate cluster(s) were defined as “ICR Medium”. The ICR high and low clusters were used to compare tumour samples with a highly or lowly active immune phenotype. Breast tumours within ICR clusters were dichotomized by median PRAME value.

### 2.2 Transcriptomic and single sample gene set enrichment analysis

We extracted *PRAME* transcriptomic data for six different cancer types (breast cancer; lung squamous cell carcinoma; colon adenocarcinoma; kidney renal clear cell carcinoma; liver hepatocellular carcinoma and skin cutaneous melanoma) and their corresponding normal tissues using TCGA Wanderer (http://maplab.imppc.org/wanderer/index.html,.^31^ TCGA breast cancer *PRAME* expression data by molecular subtype was retrieved using the Breast Cancer Integrated Platform (BCIP) (http://www.omicsnet.org/bcancer/database,^32^). Single sample gene set enrichment analysis was conducted using GSVA package (v.1.34,^33^) to compare enrichment of *PRAME* expression in cell lines and TCGA breast cancer datasets. RNA was extracted from the MDA‐MB‐468 NC and OV cell lines as described below and samples were subjected to RNA sequencing, following a quality check with QUBIT, using the SMARTer Stranded Total RNA‐Seq Kit v2 ‐ Pico Input (Takara) kit and Illumina HiSeq 4000 instrument. Quality control of the raw sequence data, trimming of adapter sequences and alignment of reads was done as previously described.^34^ Statistical analysis was performed using ANOVA with post hoc Tukey test.

### 2.3 Cell culture

The breast cancer cell line MDA‐MB‐468 was purchased from the American Tissue Culture Collection (ATCC) and authenticated by short tandem repeat analysis. MDA‐MB‐468 cells were maintained in Dulbecco's Modified Eagle's Medium (Gibco‐BRL) supplemented with 10% (v/v) foetal bovine serum (Hyclone US origin, GE Lifescience), 50 U/ml penicillin and 50 μg/ml streptomycin (Gibco‐BRL) at 37°C, 5% CO2. Regular mycoplasma testing was performed using a PCR‐based assay. HLA typing of the MDA‐MB‐468 cell line was obtained for nine different HLA loci (A, B, C, DRB1, DRB3/4/5, DQA1, DQB1, DPA1, and DPB1) with two field resolution (Table S1).

### 2.4 PRAME breast cancer cell line model

The methodology for PRAME overexpression in the MDA‐MB‐468 cell line has been described previously.^11^ Transient silencing of *PRAME* was conducted using a pool of PRAME‐specific siRNA (siGENOME SMARTpool, M‐012188‐00‐0010, Dharmacon) or scrambled control siRNA (siGENOME Non‐Targeting siRNA Pool#1, D‐001206‐13‐20, Dharmacon) and DharmaFect transfection reagent (Dharmacon), according to manufacturer's guidelines.

### 2.5 RNA isolation and cDNA synthesis

Total RNA was isolated using the PureLink RNA Mini kit (Ambion) following manufacturer's instructions. RNA concentration and quality were assessed using the Nanodrop 2000 spectrophotometer (Thermo Fisher Scientific). Reverse transcription of 1 μg RNA was performed using MMLV‐Superscript reverse transcriptase (Thermo Fisher Scientific) and random hexamers, resulting in a final concentration of 50 ng/μl cDNA.

### 2.6 Quantitative real‐time RT‐PCR

Gene expression analysis was conducted using the synthesized cDNA and specific 5′FAM‐3′MGB Taqman gene expression primer/probe sets for PRAME (Hs01022301_m1, Applied Biosystems) using the Quant Studio 7 system (Applied Biosystems). Expression levels were normalized to the reference gene *RPLPO* (4333761F, Applied Biosystems).

### 2.7 Western blotting

Protein lysates were isolated from cancer cells using RIPA buffer (Pierce) supplemented with HALT protease and phosphatase inhibitor cocktail (Thermo Fisher Scientific). Western blotting was performed according to standard protocols, as described previously.^11^ Primary antibodies include rabbit anti‐PRAME (Thermo Fisher Scientific, #PA5‐1367, 1:500) and rabbit anti‐β‐actin (Cell Signaling technologies, clone 13E5, #4970, 1:1000), followed by labelling with horseradish peroxidase‐conjugated secondary anti‐rabbit antibodies (Jackson ImmunoResearch, 1:5000). Membranes were visualized using SuperSignal™ ‐West Femto ECl substrate (Pierce) and the ChemiDoc XRS+Imaging system (Biorad). Image acquisition and densitometry analysis were performed using the Image Lab software (Biorad).

### 2.8 Peripheral blood lymphocyte isolation and activation

Buffy coats were collected from HLA‐matched healthy donors at the Hamad Medical Corporation Blood Donation Center, Qatar, in accordance with the guidelines of the Declaration of Helsinki. The study protocol was approved by the Institutional Review Board of Qatar Biomedical Research Institute (IRB #2016‐002) and the Hamad Medical Corporation (IRB #17132/17). Buffy coat samples were diluted with Dulbecco's Phosphate‐Buffered Saline (Gibco‐BRL), layered on Lymphoprep™ (Stem Cell Technologies) and subjected to density gradient centrifugation to isolate the peripheral blood mononuclear cells (PBMCs). The PBMCs were washed and frozen in liquid nitrogen in freezing media (50% FBS, 40% serum‐free Roswell Park Memorial Institute 1640 medium (RPMI), 10% Dimethyl sulfoxide) until further use. Prior to assays, PBMCs were defrosted in RPMI medium supplemented with 10% FBS and incubated overnight at 37°C and 5% CO_2_. To isolate the non‐adherent peripheral blood lymphocyte population from the PBMCs, the cells were plated in a flat‐bottom multi‐well plate (Thermo Fisher Scientific, Nunclon Δ Surface) and incubated for 2 h at 37°C and 5% CO_2_. Next, the non‐adherent PBLs were activated overnight using 2 μg/ml of plate‐bound anti‐human CD3 and CD28 antibodies (eBioscience) at 37°C and 5% CO_2_. HLA typing of PBMCs was obtained for nine different HLA loci (A, B, C, DRB1, DRB3/4/5, DQA1, DQB1, DPA1, and DPB1) with one or two field resolution and donors with matched loci to MDA‐MB‐468 were selected for further analysis (Table S2).

### 2.9 Indirect co‐culture

A total of 5 × 10^4^ cancer cells were seeded per well in a 24‐well plate. Next, activated PBLs were placed on top using transwell inserts with 0.4 µm pore size (Corning) and a Target:Effector (T:E) ratio of 1:20 to enable exchange of soluble factors between cancer cells and PBLs without direct cell‐cell contact. Wells with PBLs alone were used as negative control. The cells were co‐cultured for 72 h at 37°C and 5% CO2, after which the PBLs and cancer cells were collected for flow cytometry analysis.

### 2.10 Direct co‐culture

Activated PBLs were seeded at 1 × 10^6^ cells per well in a U‐bottom 96‐well plate. Cancer cells were labelled with 10 nM Qtracker™ −655 (Thermo Fisher Scientific) according to manufacturer's instructions. A total of 2x10^3^ cancer cells were added to the wells with activated PBLs (T:E ratio 1:50) and co‐cultured at 37°C, 5% CO_2_. After 4 h of direct co‐culture, the cells were harvested for interferon (IFN)‐γ ELISpot assay and cytotoxicity analysis by flow cytometry. In addition, 72 h direct co‐cultures of cancer cells and PBLs at T:E ratio of 1:20 were performed for immune checkpoint flow cytometry. The co‐culture supernatants were collected and stored at −80°C for Luminex array analysis.

### 2.11 ELISpot

The Human IFN‐γ ELISpot PLUS kit (HRP, Mabtech) was utilized according to manufacturer's instructions. A total of 5 × 10^4^ PBLs from short‐term (4 h) direct and long‐term (72 h) indirect co‐culture experiments were seeded per well. PBLs activated with 2 μg/ml of plate‐bound anti‐human CD3 and CD28 antibodies (eBioscience) or vehicle served as positive and negative controls respectively. The wells were incubated at 37°C, 5% CO2 for 24 h and the number of spots were quantified using an ELISpot reader (Autoimmun Diagnostika GmbH).

### 2.12 Cytotoxicity analysis

After 4hr direct co‐culture, cells were washed and resuspended in PBS, followed by staining with 7‐Aminoactinomycin D (7‐AAD) (eBiocience) at room temperature for 5 min. Flow cytometry was performed by recording 50 000 events/sample using the LSRFortessa X‐20 instrument and FlowJo V10.7.1 software (BD Biosciences). Non‐viable cancer cells were gated as positive for Qtracker and 7‐AAD staining.

### 2.13 Flow cytometric analysis of immune checkpoint expression

PBLs and cancer cells from direct and indirect co‐cultures were used for multi‐marker flow cytometry to detect the expression of immune checkpoint markers. Cells were washed and resuspended in 100 μl of staining buffer containing Human Fc Block™ (564219, BD Biosciences). The expression of immune checkpoint receptors was determined using the following antibodies: PD‐1 PE‐Dazzle 594 (329940, Biolegend), VISTA BV421 (566750, BD Biosciences), CTLA‐4 BV786 (563931, BD Biosciences), TIM‐3 BV650 (565565, BD Biosciences), LAG‐3 PE (565617, BD Biosciences), TIGIT BUV395 (747845, BD Biosciences) and CD8 BV510 (563919, BD Biosciences). In parallel, we determined the expression of respective ligands: CD80 BV510 (740150, BD Biosciences), CD86 Alexa700 (564544, BD Biosciences), PD‐L1 PE‐Cy7 (558017, BD Biosciences), PD‐L2 BV786 (563843, BD Biosciences), VISTA BV421 (566750, BD Biosciences), MHC‐II BV650 (564231, BD Biosciences), GAL‐9 PE (565890, BD Biosciences) and PVR BUV395 (748272, BD Biosciences). Flow cytometry was performed by recording 50 000 events/sample using the LSRFortessa X‐20 instrument and FlowJo V10.7.1 software (BD Biosciences).

### 2.14 Anti‐PD‐L1 treatment of direct co‐cultures

Cancer cells were seeded overnight at 1 × 10^5^ cells per well in a 48 well plate, followed by direct co‐culture with activated PBLs (T:E ratio 1:20) and treatment with 0.5 µg/ml anti‐PD‐L1 mAb atezolizumab (#A1306, Biovision, USA) or vehicle. After 24 h, cancer cells were harvested for cytotoxicity and PD‐L1 expression analysis by flow cytometry.

### 2.15 Immune pathway RT2 Profiler™ PCR array

The Cancer Inflammation and Immunity Crosstalk RT2 profiler PCR array (Qiagen, PAHS‐090) was utilized to identify differential expression of immune‐related genes in cancer cells. cDNA was synthesized from cancer cell‐derived RNA. Data was analysed using the online RT2 Profiler PCR Array Data Analysis Tool (Qiagen). Expression was normalized to the housekeeping gene *RPLPO* and the threshold value for differential expression was defined as absolute log2(FC) ≥2.

### 2.16 Cytokine array

Proteome Profiler^TM^ Human XL Cytokine Array Kit, (R&D systems, #ARY022B) was utilized to determine the relative protein levels of 101 cytokines. Cancer cells were seeded at 2.5 × 10^6^ cells in 25 cm^2^ cell culture flasks, and serum‐deprived for 48 h once 70%‐80% confluency was obtained. Conditioned media was collected and centrifuged at 2000 rpm, 4°C for 10 min to remove cell debris, and the cytokine array was performed according to manufacturer's instructions.

### 2.17 Luminex magnetic array

Cell culture supernatants from 72hr direct co‐cultures were diluted 1:2 and used to determine the expression of 39 soluble proteins with a custom Luminex array (R&D Systems, Table S3) using the Bioplex‐200 system (Biorad). A 13‐point standard curve was generated to extrapolate the soluble protein levels and the data was analysed using the Bioplex Manager Software (Biorad).

### 2.18 Gene Ontology enrichment analysis

Gene ontology (GO) enrichment analysis was performed using the PANTHER (Protein Analysis Through Evolutionary Relationships) online tool (http://www.pantherdb.org). The overrepresentation Fisher's Exact test with Bonferroni correction was utilized to identify the enriched GO biological process with *p*  ≤  0.05 and fold enrichment >50.

### 2.19 Boyden Chamber transwell migration assay

Activated PBLs were labelled with calcein (1:1000) for 30 min and added to transwell inserts (5 µm pore size, Corning) with 48hr cancer cell‐conditioned media in the lower chamber as chemoattractant. Labelled activated PBLs with serum‐free DMEM alone served as a negative control. The cells were left to migrate for 3 h at 37°C, 5% CO2. The migratory ability of the PBLs was determined by measuring the calcein relative fluorescence units (RFU) from the lower chamber using the Promega Glomax MULTI detection system (Blue Filter, Ex/Em 490/510‐570). A serial dilution of labelled PBLs was used to generate a standard curve, and the equation of the best curve fit (R^2^ > 0.9) was applied to determine the number of migrated PBLs.

### 2.20 Ibidi Chamber chemotaxis assay

We used μ‐slides (Ibidi) to determine the chemotactic ability of PBLs towards cancer cell‐conditioned media, following the manufacturer's instructions. Briefly, activated PBLs in 0.1% bovine serum albumin/RPMI were embedded in 3D rat tail collagen gel type I, and a concentration gradient of 48 h cancer cell‐conditioned media was generated. Time‐lapse live‐cell imaging was conducted at 60 s intervals for a total of 3 h (180 time points) using the Zeiss Axioimager and ZEN software (10x objective). Image J software with the manual tracking plugin was used to track cell movement within the slide channel, and X/Y coordinates were calibrated to one pixel resolution. The cell tracking coordinates were analysed using the Ibidi Chemotaxis and Migration tool software (Ibidi, v2.0).

### 2.21 Statistical analysis

Normal distribution of data was assessed using the Shapiro‐Wilk test. Parametric statistical analyses were conducted using paired Student's *t*‐test or one‐way analysis of variance (ANOVA). Data are represented as mean ± standard error of mean (SEM) of at least three independent biological replicates. Statistical analyses and data visualization were performed using GraphPad Prism v8.0.0.

## 3 RESULTS

### 3.1 PRAME expression is negatively associated with breast cancer survival

**FIGURE 1:**
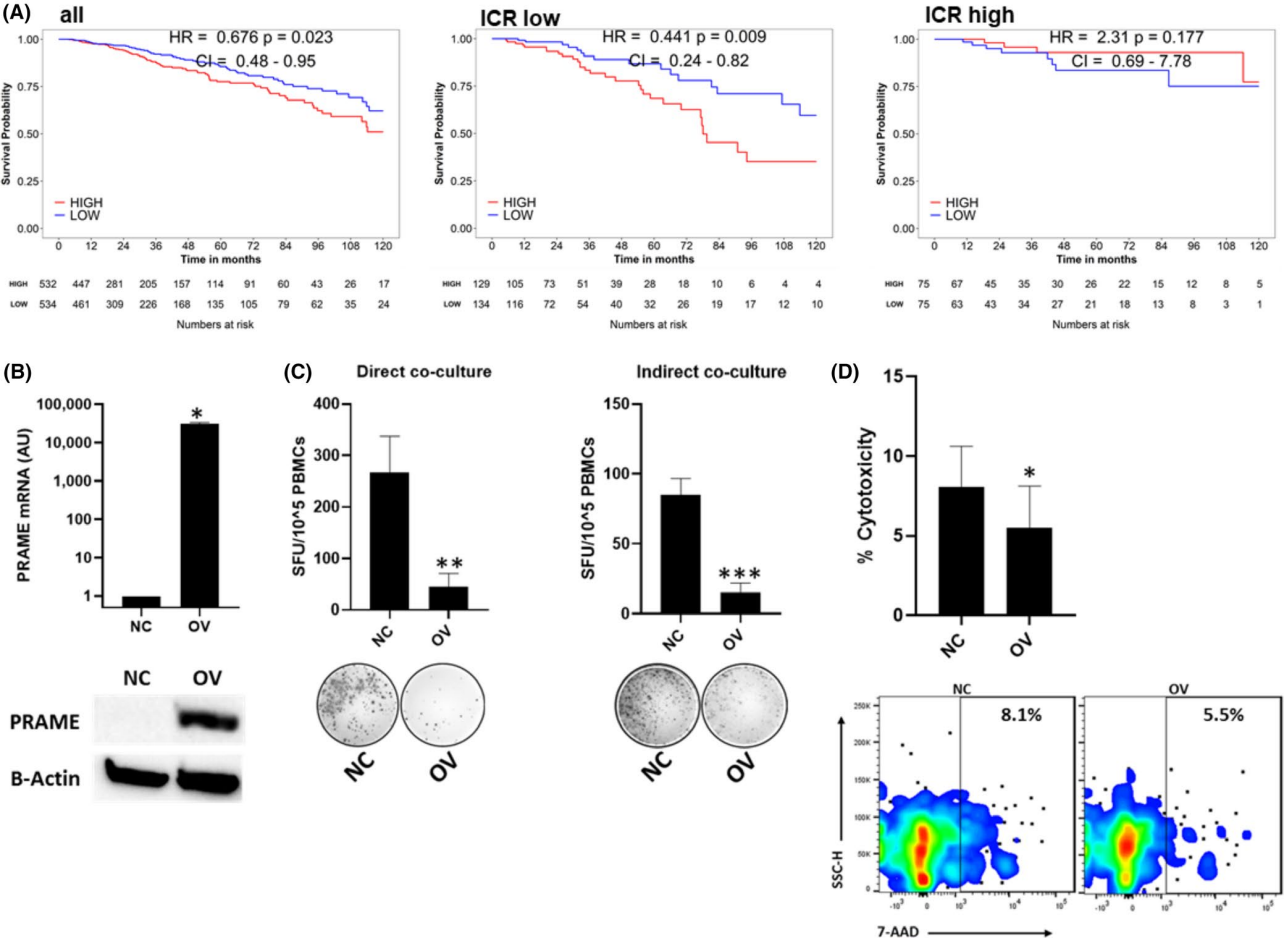
PRAME expression in breast cancer correlates with poor survival and abrogated T cell function. A, Kaplan‐Meier survival analysis of the TCGA breast cancer cohort (BRCA) based on *PRAME* expression in the total cohort (left panel), Immune Score of Rejection (ICR) low subgroup (middle panel) and ICR high subgroup (right panel). Patients were classified into subgroups with low or high *PRAME* expression based on median *PRAME* mRNA expression. B, Establishment of stable gain‐of‐function PRAME (OV) cells using breast cancer cell line MDA‐MB‐468, compared to negative control vector transfected cells (NC). Confirmation of PRAME overexpression by real‐time qRT‐PCR and western blotting (representative for 3 independent experiments). qRT‐PCR data normalized to reference gene *RPLPO*. β‐actin protein expression is depicted as a loading control. Combined data from 3 independent experiments, each performed with 1 biological replicate. C, Direct and indirect co‐culture of NC/OV cells with T cells for 72hr, followed by IFN‐γ ELISpot (*n* = 3 for each group). D, Cell viability flow cytometry after direct co‐culture of NC/OV with T cells using 7‐AAD and QTracker labelling dye (*n* = 3 for each group). Non‐viable cancer cells were gated as positive for Qtracker and 7‐AAD staining. IFN‐γ ELISpot images and cell viability flow cytometry plots are given for one donor, representative for 3 independent experiments. Combined data from 3 independent experiments, each performed with one donor (biological replicate), are shown. Bars indicate mean with standard error of mean (±SEM). All statistical analysis performed using paired Student's *t*‐test. *p ≤ 0.05, **p ≤ 0.01, ***p ≤ 0.001. NC, negative control; OV, PRAME overexpression

Based on the increased expression of PRAME in breast tumours, especially basal‐like and luminal B tumours (Figure S1A‐B), we interrogated the TCGA breast cancer cohort to assess the prognostic connotation of PRAME tumour expression (Figure 1A). High PRAME tumour expression was associated with shorter overall survival (HR = 0.676, *p* = 0.023). Furthermore, immune‐unfavourable breast tumours, defined as ICR low by the ICR classifier,^35^ could be stratified based on PRAME tumour expression, whereby patients with a higher PRAME expression experienced worse survival (HR = 0.441, *p* = 0.009). Of note, PRAME dichotomization did not further stratify immune favourable (ICR high) tumours, suggesting that PRAME may be involved in regulating an immunosuppressive tumour microenvironment.

### 3.2 PRAME‐expressing cancer cells dampen T cell activation and cytolytic ability

Given the prognostic connotation of PRAME expression in immune‐unfavourable breast tumours, we sought to investigate the role of PRAME in modulating the tumour immune microenvironment and the anti‐tumour immune response. For this purpose, we developed a breast cancer cell line model with stable overexpression of PRAME using the MDA‐MB‐468 basal‐like/triple negative breast cancer cell line (Figure 1B). Comparative analysis of PRAME RNAseq expression in our MDA‐MB‐468 cell line model with the expression in TCGA breast cancer samples, and in particular TNBCs (Figure S1C), confirmed the physiological relevance of the PRAME expression levels in the overexpressing cell line.

**FIGURE 2:**
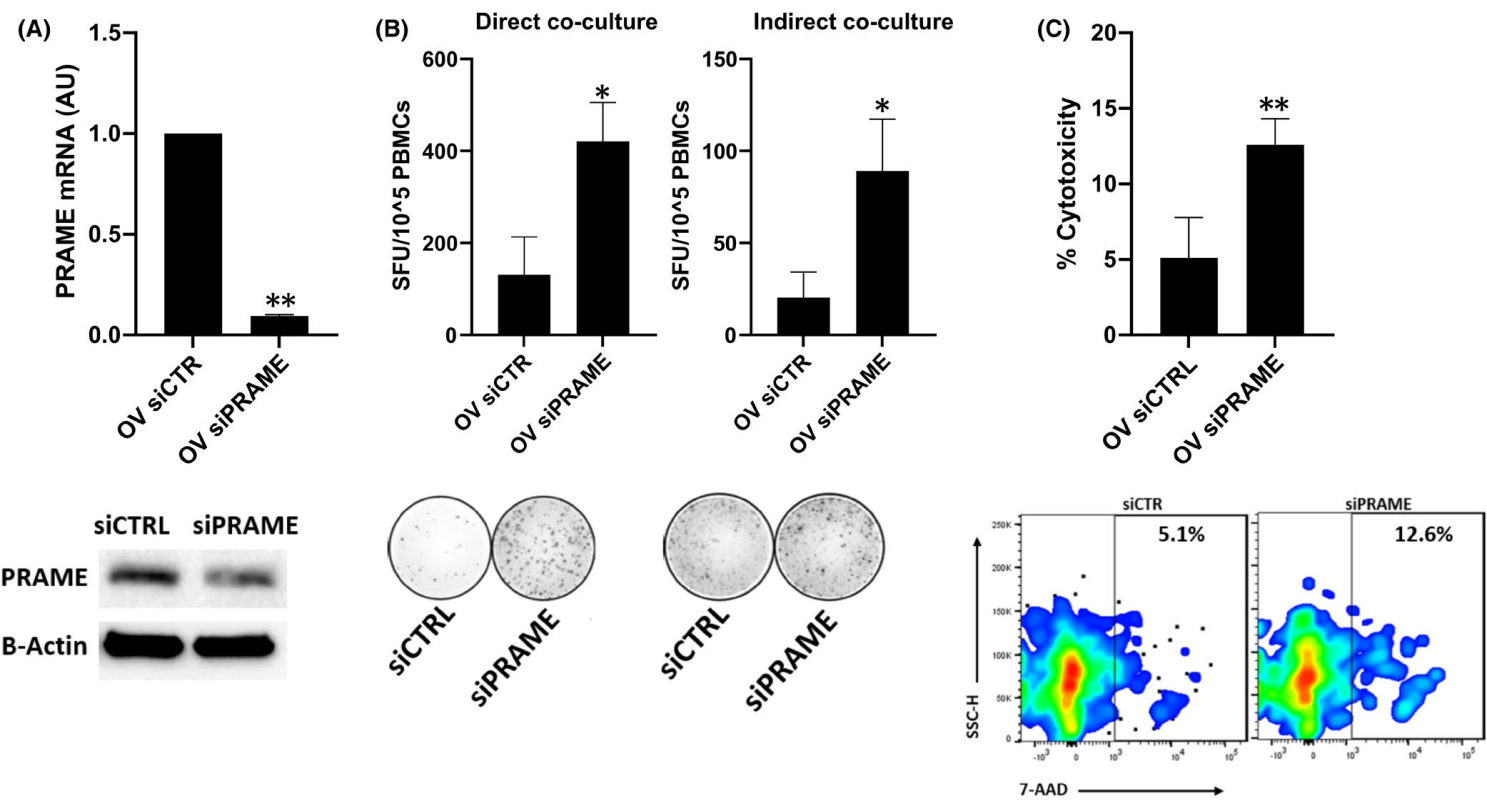
PRAME silencing improves T cell activation and cytotoxicity. A, Establishment of transient PRAME knockdown model (siPRAME) using PRAME overexpressing MDA‐MB‐468 cells, compared to negative scrambled siRNA transfected cells (siCTRL). Confirmation of PRAME knockdown by real‐time qRT‐PCR and western blotting (representative of 3 independent experiments). qRT‐PCR data normalized to reference gene *RPLPO*. β‐actin protein expression is depicted as a loading control. Combined data from 3 independent experiments, each performed with 1 biological replicate, are shown. B, Direct and indirect co‐culture of siCTRL/siPRAME cells with HLA‐matched T cells, followed by IFN‐γ ELISpot (*n* = 3 for each group). C, Cell viability flow cytometry after direct co‐culture using 7‐AAD and QTracker labelling dye (*n* = 3 for each group). Non‐viable cancer cells were gated as positive for Qtracker and 7‐AAD staining. IFN‐γ ELISpot images and cell viability flow cytometry plots are given for one donor, representative for 3 independent experiments. Combined data from 3 independent experiments, each performed with one donor (biological replicate), are shown. Bars indicate mean with standard error of mean (±SEM). All statistical analysis performed using paired Student's *t*‐test. *p ≤ 0.05, **p ≤ 0.01

Next, we performed co‐culture experiments to determine the effect of PRAME overexpressing cancer cells on the activity and function of HLA‐matched T cells. Interestingly, T cell activation, as determined by IFN‐γ secretion, was reduced in the presence of PRAME overexpressing cells in both direct (*p* ≤ 0.01) and indirect (*p* < 0.001) co‐cultures, suggesting that different mechanisms may be involved (Figure 1C). Moreover, PRAME overexpressing cells dampened (*p* ≤ 0.05) cytolytic potential, as observed by a decrease in cancer cell death following direct co‐culture (Figure 1D). These observations were reversible by silencing of *PRAME* at the mRNA and protein expression level (Figure 2A). Silencing of *PRAME* significantly increased T cell activation (*p* < 0.05, Figure 2B), and cytolytic potential (*p* < 0.01, Figure 2C) in direct and indirect co‐cultures.

### 3.3 PRAME expression is associated with immune checkpoint expression

**FIGURE 3:**
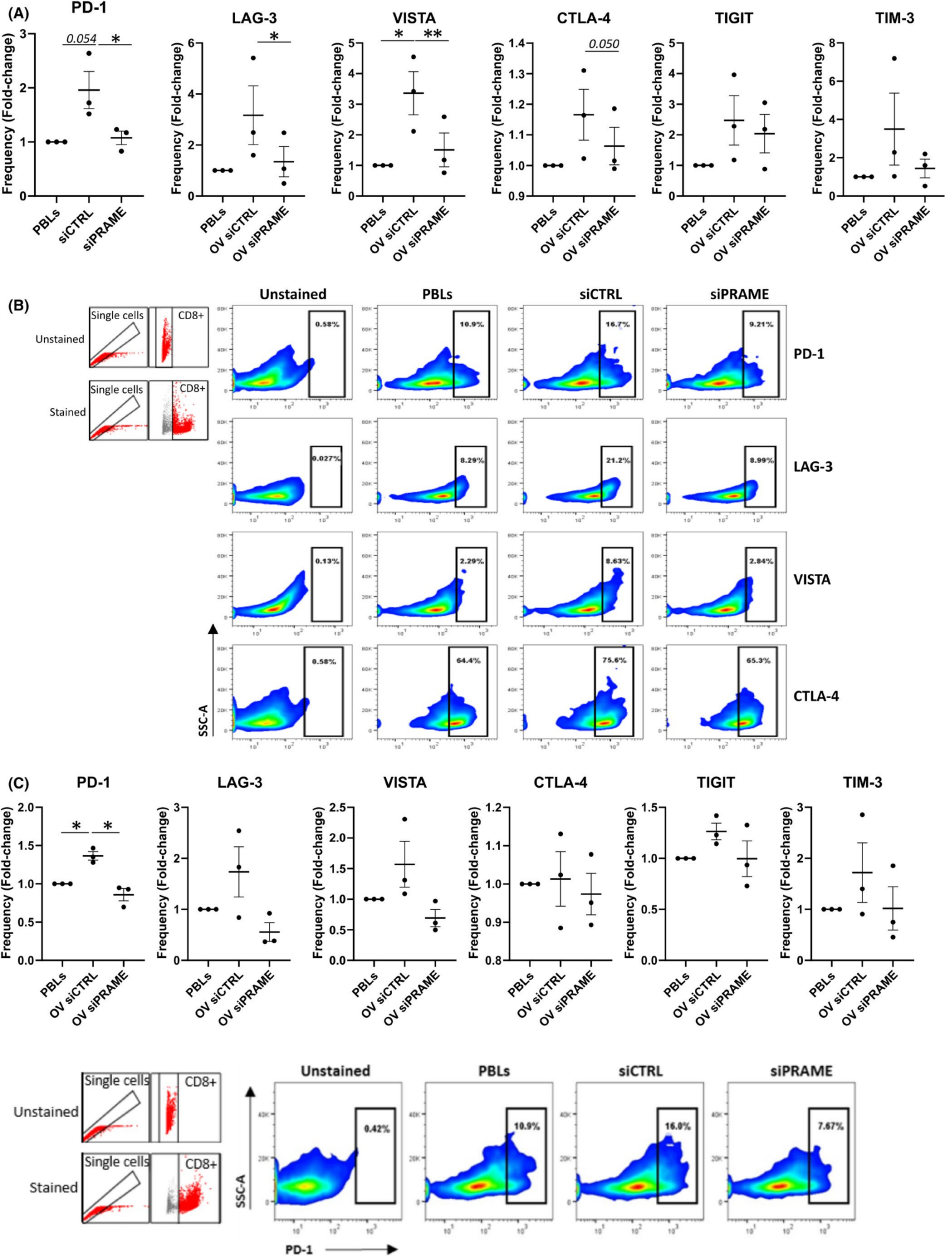
Co‐culture with PRAME‐silenced cancer cells decreases immune checkpoint expressing T cells. Multi‐parameter flow cytometry was conducted to assess the frequency of HLA‐matched CD8+ T cells expressing PD‐1, LAG‐3, VISTA, CTLA‐4, TIGIT and TIM‐3 immune checkpoint proteins after co‐culture with cancer cells for 72h. A, Mean fold‐change in frequency of CD8+ T cells expressing respective immune checkpoints after direct co‐culture with siCTRL or siPRAME cells relative to PBLs alone (*n* = 3 for each group). B, Flow cytometry plots of CD8+ subpopulation from one donor, representing 3 independent experiments. Ancestral plots with single cells and CD8+ gating strategies are indicated at the top left. C, Mean fold‐change in frequency of CD8+ T cells expressing respective immune checkpoints after indirect co‐culture with siCTRL or siPRAME cells relative to PBLs alone (*n* = 3 for each group). D, Flow cytometry plots of PD‐1+ CD8+ subpopulation for one donor, representing 3 independent experiments. Ancestral plots with single cells and CD8+ gating strategies are indicated on the left. Combined data from 3 independent experiments, each performed with one donor (biological replicate), are shown. Bars indicate mean with standard error of mean (±SEM). Statistical analysis performed using paired Student's *t*‐test (PBLs vs siCTRL, siCTRL vs siPRAME). *p ≤ 0.05, **p ≤ 0.01. NC, negative control; OV, overexpression

The significant, reversible reduction in T cell activation and cancer cell killing in direct contact with PRAME overexpressing cancer cells prompted us to investigate whether silencing of *PRAME* alleviates this immunosuppressive effect by altering the immune checkpoint repertoire, key mediators of direct cell‐cell T cell activation. Using flow cytometry, we determined the CD8+ T cell expression of multiple immune checkpoints, including PD‐1, LAG‐3, VISTA, CTLA‐4, TIGIT and TIM‐3 (Figure 3). Upon co‐culture with PRAME overexpressing cancer cells (siCTRL), the frequency of CD8+ T cells expressing individual immune checkpoints increased. Furthermore, silencing of *PRAME* restored the proportion of CD8+ T cells expressing PD‐1 (*p* < 0.05), LAG‐3 (*p* < 0.05), VISTA (*p* < 0.01) and CTLA‐4 (*p* = 0.05) to baseline levels, while a trend towards a reduced frequency was observed for TIGIT and TIM‐3 (Figure 3A‐B). Interestingly, a similar decrease was observed in the frequency of PD‐1 positive CD8+ T cells (*p* < 0.05) using indirect co‐cultures with *PRAME*‐silenced cancer cells (Figure 3C‐D). Thus, PRAME expression in our breast cancer cell line model can modulate the immune checkpoint expression on CD8+ T cells, most prominently in direct contact co‐cultures but also through indirect co‐culture. We did not detect double immune checkpoint CD8+ T cell populations expressing CTLA4+/PD1+ or CTLA4+/LAG3+ in the presence of either cell line model (data not shown). We did identify a CTLA4+/TIM3+ CD8+ T cell subset, however, no significant changes in its frequency were observed in the presence of PRAME overexpressing cancer cells (data not shown).

**FIGURE 4:**
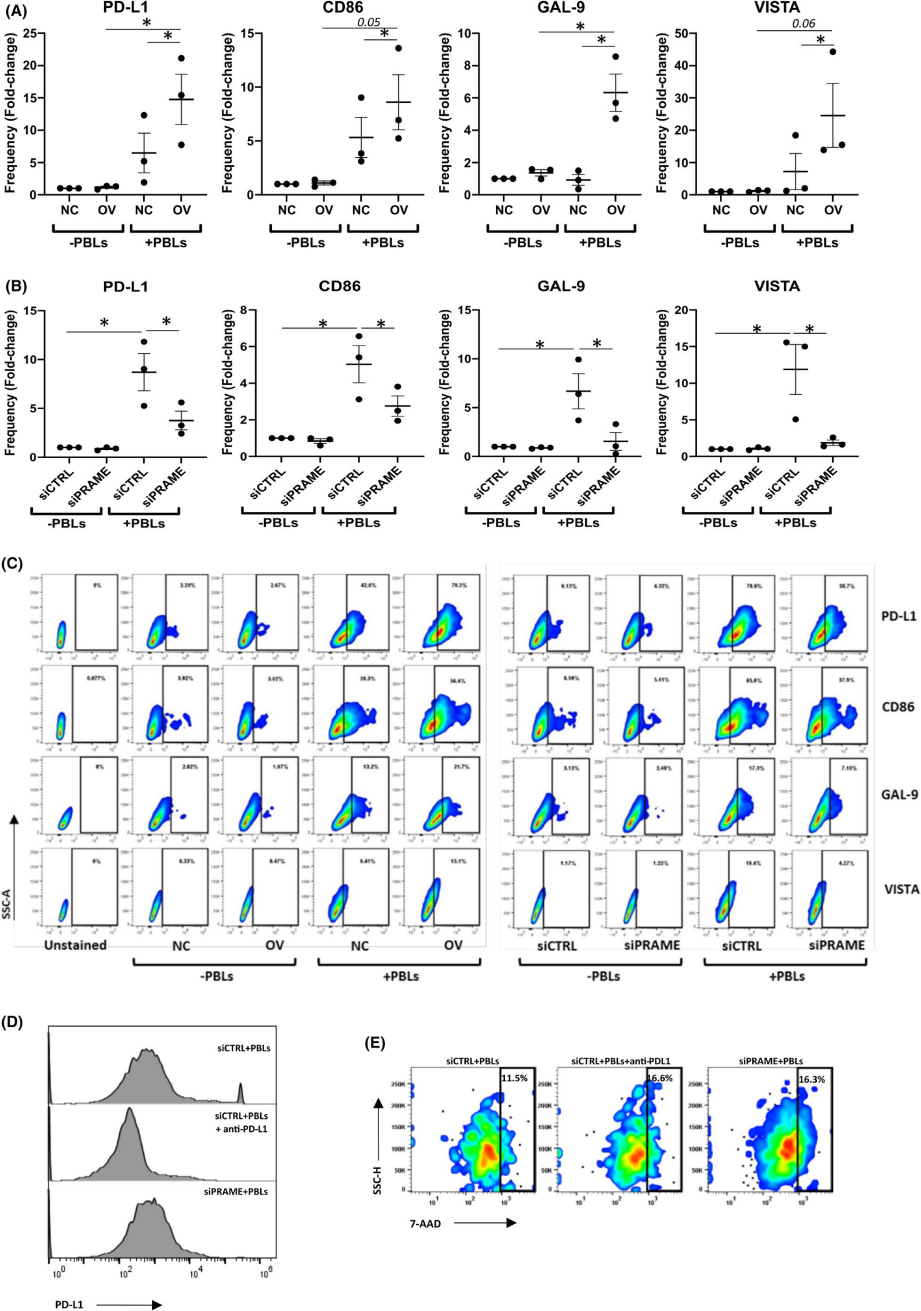
PRAME expression modulates immune checkpoint ligand expression and restores cancer cell killing ability to levels seen with PD‐L1 treatment. Multi‐parameter flow cytometry was conducted to assess the frequency of cancer cells expressing PD‐L1, CD86, GAL‐9 and VISTA immune checkpoint ligands after indirect co‐culture (72 h). A, Mean fold‐change in frequency of NC/OV cancer cells expressing respective immune checkpoint ligands (*n* = 3 for each group). Fold‐change is relative to frequency of NC cells‐PBLs. B, Mean fold‐change in frequency of siCTRL/siPRAME cancer cells expressing respective immune checkpoint ligands (*n* = 3 for each group). Fold‐change is relative to frequency of siCTRL cells ‐PBLs. C, Flow cytometry plots of single cell gated cancer cells in co‐culture with PBLs from one donor, representing 3 independent experiments. D, PD‐L1 expression analysis by flow cytometry after treatment with atezolizumab (*n* = 2 for each group). E, Cell viability flow cytometry using 7‐AAD and QTracker labelling dye after treatment with atezolizumab (*n* = 2 for each group). Non‐viable cancer cells were gated as positive for Qtracker and 7‐AAD staining. Combined data from 3 independent experiments, each performed with one donor (biological replicate), are shown in panels A, B and C. Panels D & E show combined data from 2 independent experiments, each performed with one donor (biological replicate). Bars indicate mean with standard error of mean (±SEM). Statistical analysis performed using paired Student's *t*‐test (NC+PBLs vs OV+PBLs, OV‐PBLs vs OV+PBLs, siCTRL+PBLs vs siPRAME+PBLs, siCTRL‐PBLs vs siCTRL+PBLs). *p ≤ 0.05. NC, negative control; OV, PRAME overexpression, PBL, peripheral blood lymphocytes

Furthermore, analysis of immune checkpoint ligand expression of cancer cells alone revealed no difference in the frequency of cells expressing PD‐L1, CD86, GAL‐9 or VISTA (Figure 4A‐C) or PD‐L2, CD80, HLA‐DR or PVR (Figure S2) in the presence of PRAME. However, upon incubation with PBLs we found an increase (*p* < 0.05) in the number of PRAME overexpressing cells expressing PD‐L1, CD86, GAL‐9 and VISTA (Figure 4A and 4C), which was reversible by silencing of *PRAME* (*p* < 0.05, Figure 4B,C). No differences were found in the frequency of CD8+ T cell subsets with double expression of immune checkpoint ligands (data not shown).

Given the dual effect of PRAME on PD‐1 and PD‐L1 expression, we next compared the effect of silencing of *PRAME* on cytotoxicity in comparison to immune checkpoint blockade with the PD‐L1 inhibitor atezolizumab (Figure 4D,E). Atezolizumab reduced PD‐L1 expression to a greater extent than *PRAME* silencing (Figure 4D). Further, atezolizumab treatment of PRAME‐expressing cell co‐cultures increased cytotoxicity (11.5% in siCTRL+PBLs vs 16.6% in siCTRL+PBLs+anti‐PDL1) (Figure 4E). Interestingly, silencing of *PRAME* alone induced cancer cell killing at similar levels (16.3 vs 16.6%, siPRAME+PBLs vs siCTRL+PBLs+anti‐PDL1). Although these changes seem modest, they potentially indicate that targeting of PRAME could help alleviate inhibition of T cell activation by immune checkpoints.

### 3.4 PRAME‐expressing cancer cells deregulate immune‐related genes and secretion of cytokines

**FIGURE 5:**
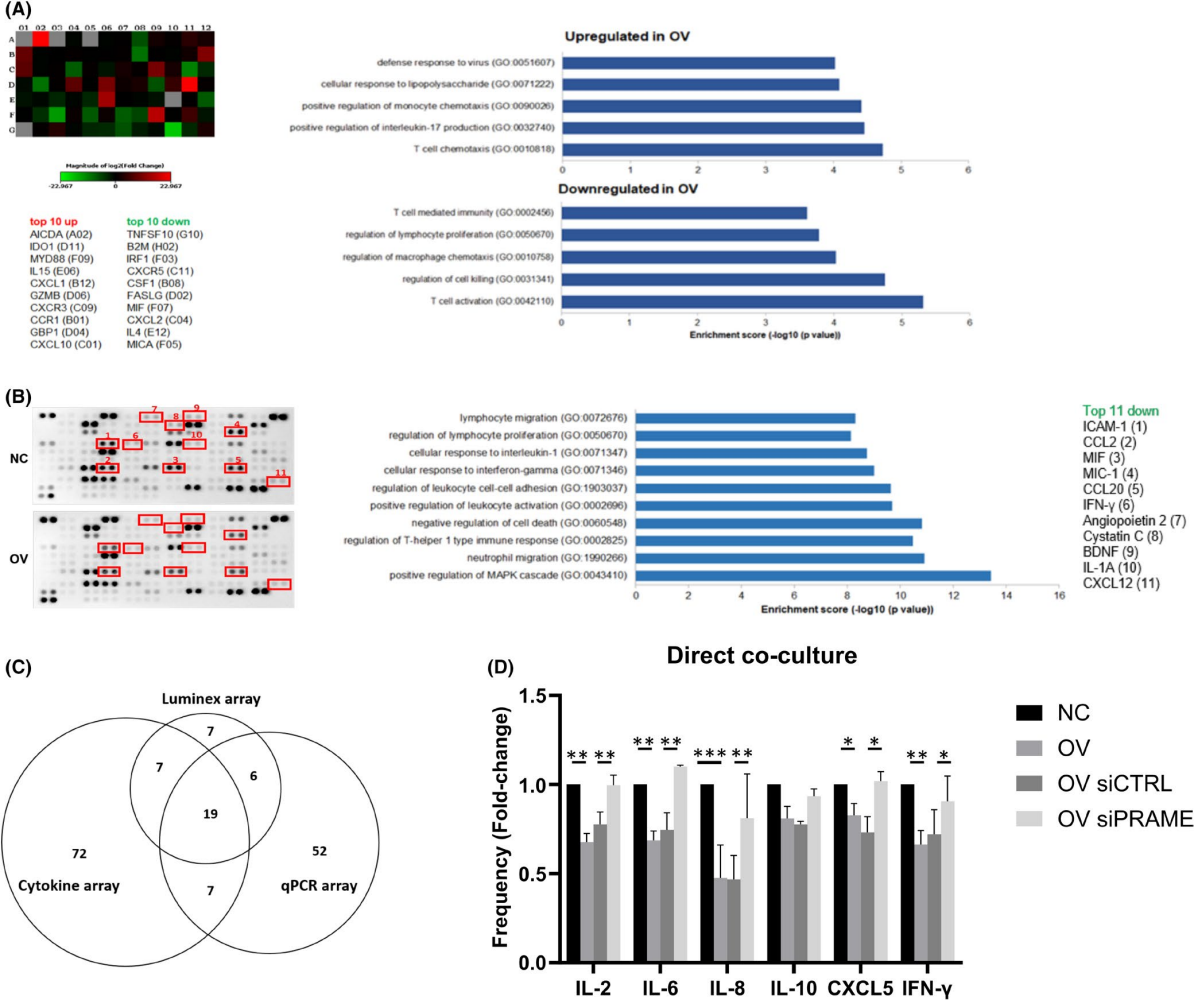
PRAME perturbs expression of immune response‐related molecules. A, RT2 Profiler Cancer Inflammation & Immunity Crosstalk qRT‐PCR array of 84 immune‐related genes in OV versus NC cells (*n* = 1 for each group with 2 technical replicates each). Representative GO enrichment analysis of top 10 differentially expressed genes. B, Cytokine protein secretion by NC/OV cells (48 h conditioned media) as determined by the Proteome Profiler protein array (105 cytokines) and GO enrichment analysis of downregulated molecules (*n* = 1 for each group with 2 technical replicates each). C, Venn diagram depicting overlapping targets quantified by the qPCR array, cytokine array and the luminex array. D, Quantification of cytokines in direct co‐culture conditioned media (72 h) using a custom luminex panel (39 cytokines). Cytokine concentrations are expressed as mean fold‐change normalized to NC sample (*n* = 3 for each group). Combined data from 3 independent experiments, each performed with one donor (biological replicate), are shown. Statistical analysis performed using paired Student's *t*‐test (NC vs OV, siCTRL vs siPRAME). * *p* ≤ 0.05, ** *p* ≤ 0.01, ***p≤0.001. NC, negative control; OV, PRAME overexpression

Since we found that PRAME overexpressing cancer cells dampen lymphocyte activation, function and immune checkpoint expression in both direct contact and indirect co‐cultures, we next investigated the effect of PRAME on the expression of immune‐related genes and cytokine secretion in cancer cells. First, we determined the mRNA expression levels of 84 inflammatory genes involved in mediating communication between tumour cells and immune cells in addition to potential oncogenic functions. Differential expression analysis of PRAME overexpressing and control cancer cells (*p* < 0.05, fold enrichment >50) identified the top 10 upregulated genes to include *AICDA*, *IDO1*, *MYD88*, *IL*‐*15*, *CXCL1*, *GZMB*, *CXCR3*, *CCR*, *GBP1* and *CXCL10*, whereas the top 10 downregulated genes include *TNFSF10*, *B2M*, *IRF1*, *CXCR5*, *CSF1*, *FASLG*, *MIF*, *CXCL2*, *IL4* and *MICA* (Figure 5A). Gene ontology analysis of the differentially expressed genes revealed an enrichment of pathways involved in the regulation of monocyte and T cell chemotaxis, microbial and viral immune response, and production of IL17. On the other hand, downregulated genes were mainly enriched in pathways involved in T cell mediated immunity, regulation of T cell activation, proliferation, cell killing and macrophage chemotaxis.

Next, we investigated whether PRAME tumour expression could alter the production of secreted proteins in order to support an immunosuppressive environment. Comprehensive analysis of the levels of 105 proteins demonstrated an overall decrease in secreted proteins by PRAME overexpressing cells with the top downregulated being ICAM‐1, CCL2, MIF, MIC‐1, CCL20, IFN‐γ, angiopoietin, cystatin C, BDNF, IL‐1A and CXCL12 (Figure 5B). Gene ontology analysis revealed that the downregulated cytokines are involved in supporting a pro‐inflammatory immune response and regulating immune cell migration and chemotaxis.

Based on these findings, we next sought to assess cytokine expression when co‐culturing our breast cancer cell line model with PBLs. To this end, we designed a customized 39‐plex immunoassay, comprising 32 molecules identified in the previous two assays of which 19 were common across the qRT‐PCR array and proteome profiler array (Figure 5C, Table S1). Co‐cultures of PRAME overexpressing cells showed a significant decrease in the expression of immunostimulatory cytokines such as IL‐2 (*p* < 0.01), IL‐6 (*p* < 0.01), IL‐8 (*p* < 0.001) and IFN‐γ (*p* < 0.01), and of the neutrophil chemokine CXCL5 (*p* < 0.05), and a non‐significant trend of reduced IL‐10 (Figure 5D). Conversely, silencing of *PRAME* restored the levels of these cytokines to that observed in control cells. Together, these results indicate that PRAME tumour expression has the potential to modulate soluble immunostimulatory and immunosuppressive factors.

### 3.5 PRAME cancer cell expression does not affect T cell chemotaxis

As we observed that PRAME overexpressing cancer cells alter the expression and secretion of several chemotaxis‐related molecules, we next investigated if conditioned media from PRAME overexpressing cancer cells could affect the chemotactic ability of PBLs. We did not find any difference in the vertical migratory ability of PBLs using conditioned media from PRAME overexpressing or control cells (Figure S3A). Similarly, assessing chemotaxis across a collagen type I‐matrix indicated random PBL movement (Rayleigh test *p*‐value <0.001) in the presence of conditioned media from either PRAME overexpressing or control cells (Figure S3B). More specifically, we show that in neither condition the lymphocytes demonstrated a change in directionality (<1.0), nor a change in forward migration indices or the centre of mass coordinates. Although these findings might seem contradictory to our protein profiling results, it is important to note that the latter demonstrated a PRAME‐associated decrease in the secretion of chemokines regulating monocyte/macrophage and neutrophil, rather than lymphocyte chemotaxis.

## 4 DISCUSSION

Cancer immunotherapy has shown great promise in inducing potent and durable immune responses against tumour cells. However, its efficacy remains limited to a minority of patients from a select number of cancer types. Plausible mechanisms for this variability in response involve alterations in cancer immunobiological molecules and pathways, tumour heterogeneity, and the presence of an immunosuppressive microenvironment.^36^ Specifically, changes in tumour intrinsic and extrinsic factors within the microenvironment have been shown to shape immunotherapy response.^37^, ^38^, ^39^, ^40^ Therefore, a better understanding of the cellular and molecular interactions within the tumour microenvironment is critical for the development of effective strategies to improve cancer immunotherapy.

Cancer testis antigens such as NY‐ESO‐1 and PRAME have been widely studied as candidate target for immunotherapy given their restricted expression patterns and immunogenic nature.^19^, ^41^ In this study, we investigated whether PRAME tumour expression could be involved in modulating anti‐tumour immunity. We demonstrated that PRAME overexpressing breast cancer cells impair T cell activity and cytotoxicity, induce immune checkpoint expression, and dysregulate inflammatory cytokine and chemokine production. In accordance, we have shown that PRAME tumour expression can further stratify breast cancer patients with immune‐unfavourable tumours (ICR low), whereby a higher PRAME expression bestows a worse prognosis thereby introducing another level of complexity in terms of immunosuppression.

Interestingly, we found that a significantly higher proportion of PRAME overexpressing cancer cells express immune checkpoint ligands such as PD‐L1, CD86, GAL‐9 and VISTA, which may in part explain the reduced T cell mediated activation and killing of PRAME overexpressing cancer cells. Our PD‐L1 observation is in contrast to the report on dedifferentiated liposarcomas where PRAME expression was negatively associated with PD‐L1 expression.^25^ This discrepancy suggests that the role of PRAME in cancer may differ depending on tissue‐ and context‐specific features and warrants further investigation. In addition, silencing of PRAME reduced the frequency of CD8+ T cells with expression of the immune checkpoints PD‐1, LAG‐3 and VISTA, indicating that PRAME tumour expression considerably impairs the PD‐L1/PD‐1 axis by dysregulating both the ligand and receptor. Upregulated expression of PD‐L1 is a common feature of tumour cells to avoid immune‐mediated destruction and blocking PD‐L1 or its receptor PD‐1 with antagonistic monoclonal antibodies is a well‐recognized approach to boost the anti‐tumour immune response in many different cancer types.^42^, ^43^, ^44^ Hence, we investigated whether targeting PRAME could be used as an approach to modulate the activity of the PD‐L1/PD‐1 pathway. Notably, we found that silencing of *PRAME* induces cancer cell killing to similar levels as inhibition of PD‐L1 using atezolizumab. As such, it would be of interest to further study the effect of PRAME tumour expression on other immune checkpoints such as TIM‐3 and LAG‐3 within the context of immune checkpoint blockade.

Interestingly, we found that PRAME expression in cancer cells modulates T cell activation through direct cell‐cell interaction as well as through indirect contact by the secretion of soluble mediators. This observation demonstrates that PRAME‐specific interventions may impact lymphocytes that are in direct contact with cancer cells or that are within the tumour microenvironment. Using three different platforms, we demonstrated PRAME‐associated dysregulation of 19 immunoregulatory factors among which the pro‐inflammatory cytokines IFN‐γ, IL‐2, IL‐6 and IL‐8, and the neutrophil chemokine CXCL5 displayed the most robust decrease. Given the important roles of these cytokines in antigen presentation, activation of effector T cell and natural killer cell proliferation and maturation, migration and infiltration of CD8+ T cells and neutrophils, it is likely that the PRAME‐associated T cell dysfunction in our model is linked to their dysregulation.^45^, ^46^, ^47^, ^48^

Collectively, our findings support a potential dual purpose for targeting of PRAME in cancer; either through direct targeting of cancer cells based on PRAME immunogenicity or by inhibiting oncogenic traits of PRAME, or through indirect disruption of PRAME‐associated immunomodulation. The notion of an immunoregulatory role for PRAME prompts us to cautiously speculate that PRAME‐based therapy in combination with immunotherapy may improve treatment response. However, this may not be the case for immune checkpoint blockade where silencing of *PRAME* reduces the a priori expression of immune checkpoints and their ligands, in particular PD‐L1 and PD‐1, thereby reducing target density for intervention with immune checkpoint inhibitors. The findings from this study remain to be confirmed in additional autologous in vitro and in vivo models, and warrant further study of the interplay between PRAME‐expressing cancer cells and immune cells within their microenvironment.

## CONFLICT OF INTEREST

The authors confirm that there are no conflicts of interest.

## AUTHOR CONTRIBUTION

**Adviti Naik:** Formal analysis (lead); Investigation (equal); Methodology (equal); Writing‐original draft (lead). **Remy Thomas:** Formal analysis (equal); Investigation (equal); Writing‐original draft (equal). **Ghaneya Al‐Khadairi:** Formal analysis (equal); Investigation (equal); Writing‐original draft (equal). **Rim Bacha:** Investigation (supporting); Writing‐original draft (supporting). **Wouter Hendrickx:** Formal analysis (supporting); Writing‐review & editing (supporting). **Julie Decock:** Conceptualization (lead); Funding acquisition (lead); Methodology (equal); Writing‐review & editing (lead).

## Supporting information

Figure S1

Figure S2

Figure S3

Table S1‐S3

## Data Availability

The data presented in this study are available on reasonable request from the corresponding author.
