# Supplementary figures and images for: Cancer testis antigen PRAME: An anti‐cancer target with immunomodulatory potential
Source: J Cell Mol Med. 2021 Oct 6;25(22):10376–88. doi: 10.1111/jcmm.16967 (PMC8581324; doi:10.1111/jcmm.16967)

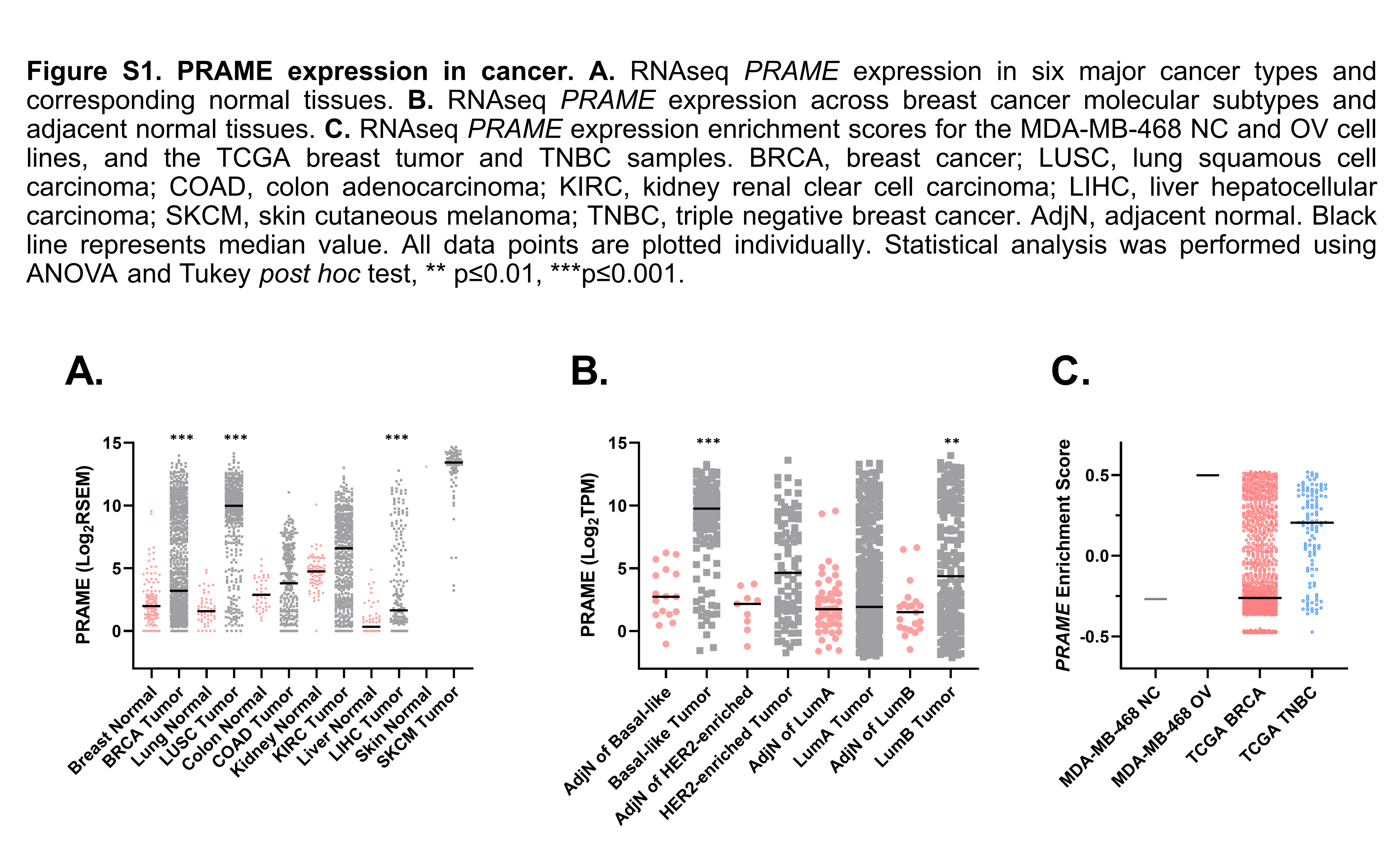

Supplement: Supplementary file 1 — Figure S1 [file JCMM-25-10376-s001.png]

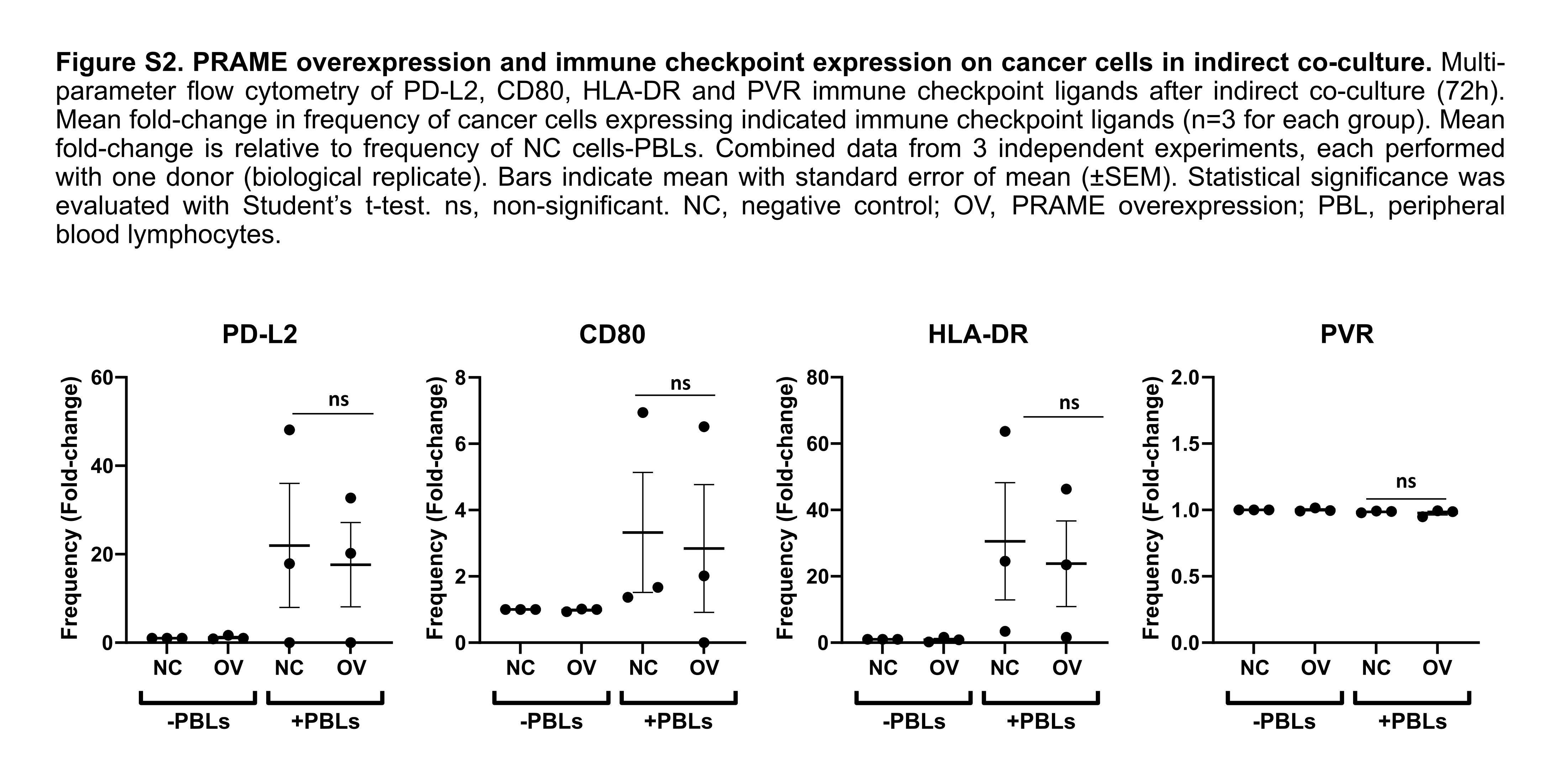

Supplement: Supplementary file 2 — Figure S2 [file JCMM-25-10376-s003.png]

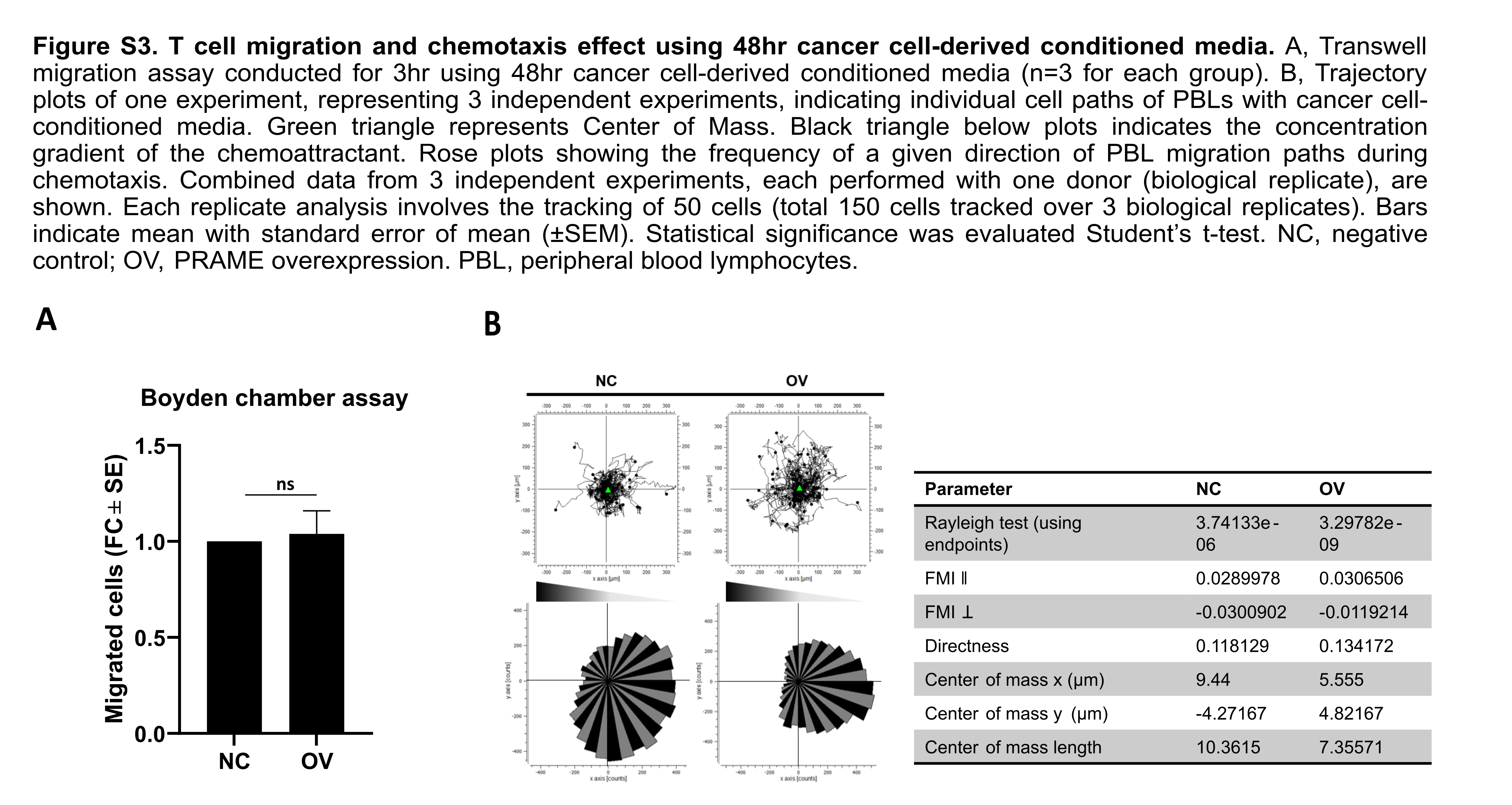

Supplement: Supplementary file 3 — Figure S3 [file JCMM-25-10376-s002.png]
