# Supplementary material for: Cancer testis antigen PRAME: An anti‐cancer target with immunomodulatory potential
Source: J Cell Mol Med. 2021 Oct 6;25(22):10376–88. doi: 10.1111/jcmm.16967 (PMC8581324; doi:10.1111/jcmm.16967)
Supplement: Supplementary file 4 — Table S1‐S3 [file JCMM-25-10376-s004.docx]

**SUPPLEMENTARY TABLES**

**Table S1. MDA-MB-468 HLA typing**

| **HLA-A** | A*23:01 | A*30:02 |
| --- | --- | --- |
| **HLA-B** | B*27:03 | B*53:01 |
| **HLA-C** | C*02:02 | C*04:01 |
| **HLA-DRB1** | DRB1*07:01 | DRB1*01:02 |
| **HLA-DRB3, 4, 5** | DRB4*01:01 | - |
| **HLA-DQA1** | DQA1*01:01 | DQA1*02:01 |
| **HLA-DQB1** | DQB1*02:02 | DQB1*05:01 |
| **HLA-DPA1** | DPA1*01:03 | DPA1*02:01 |
| **HLA-DPB1** | DPB1*01:01 | DPB1*29:01 |

**Table S2. Donor HLA match**

| **Donor ID** | **HLA loci match with MDA-MB-468** |
| --- | --- |
| **BC03** | C*04, DPB4*01, DPA1*01 |
| **BC04** | A*23, DRB1*07, DPB4*01, DQB1*01, DPA1*01 |
| **BC07** | C*04, DRB1*01, DQA1*01, DPA1*01 |
| **BC10** | A*23, B*27, C*04, DQA1*01, DQB1*05, DPA1*01 |
| **BC15** | DRB1*07:01, DQA1*02:01, DQB1*02:02, DPA1*01:03 |

**Table S3. Luminex Human Magnetic Custom Array (39-Plex)**

| **Analyte** | **Bead Region** |
| --- | --- |
| CCL2/JE/MCP-1 | 62 |
| CCL3/MIP-1 alpha | 35 |
| CCL4/MIP-1 beta | 37 |
| CCL7/MCP-3/MARC | 19 |
| CCL21/6Ckine | 65 |
| CCL22/MDC | 36 |
| CD27/TNFRSF7 | 67 |
| CD40/TNFRSF5 | 76 |
| CD40 Ligand/TNFSF5 | 74 |
| CXCL1/GRO alpha/KC/CINC-1 | 77 |
| CXCL2/GRO beta/MIP-2/CINC-3 | 27 |
| CXCL5/ENA-78 | 47 |
| CXCL9/MIG | 52 |
| CXCL10/IP-10/CRG-2 | 21 |
| EGF | 25 |
| Fas Ligand/TNFSF6 | 39 |
| Galectin-1 | 51 |
| Galectin-3 | 34 |
| GITR/TNFRSF18 | 61 |
| GM-CSF | 46 |
| Granzyme B | 57 |
| IFN-gamma | 29 |
| IL-1 alpha/IL-1F1 | 38 |
| IL-1 beta/IL-1F2 | 28 |
| IL-2 | 43 |
| IL-3 | 15 |
| IL-4 | 75 |
| IL-5 | 53 |
| IL-6 | 13 |
| IL-7 | 20 |
| IL-8/CXCL8 | 18 |
| IL-10 | 22 |
| IL-12 p70 | 56 |
| IL-13 | 64 |
| IL-15 | 63 |
| IL-17/IL-17A | 42 |
| M-CSF | 48 |
| TNF-alpha | 12 |
| TRAIL/TNFSF10 | 72 |
